# Delimiting shades of gray: phylogeography of the Northern Fulmar, *Fulmarus glacialis*

**DOI:** 10.1002/ece3.597

**Published:** 2013-05-22

**Authors:** Kevin C R Kerr, Carla J Dove

**Affiliations:** Division of Birds, National Museum of Natural History, Smithsonian InstitutionWashington, DC, 20560

**Keywords:** Arctic, Atlantic, DNA barcodes, MC1R, Pacific, polymorphism

## Abstract

The Northern Fulmar (*Fulmarus glacialis*) is a common tube-nosed seabird with a disjunct Holarctic range. Taxonomic divisions within the Northern Fulmar have historically been muddled by geographical variation notably including highly polymorphic plumage. Recent molecular analyses (i.e., DNA barcoding) have suggested that genetic divergence between Atlantic and Pacific populations could be on par with those typically observed between species. We employ a multigene phylogenetic analysis to better explore the level of genetic divergence between these populations and to test an old hypothesis on the origin of the modern distribution of color morphs across their range. Additionally, we test whether mutations in the melanocortin-1 receptor gene (MC1R) are associated with dark plumage in the Northern Fulmar. We confirmed that mitochondrial lineages in the Atlantic and Pacific populations are highly divergent, but nuclear markers revealed incomplete lineage sorting. Genetic divergence between these populations is consistent with that observed between many species of Procellariiformes and we recommend elevating these two forms to separate species. We also find that MC1R variation is not associated with color morph but rather is better explained by geographical divergence.

## Introduction

Drawing species limits between allopatric populations is a long-standing problem in biology (Mayr 1963). However, this endeavor has been greatly facilitated in recent decades by the application of molecular methods, which yield data that are less susceptible to homoplasy and inform divergence estimates. Attempts to streamline this molecular approach to taxonomy and to generally address the “taxonomic impediment” contributed to the advent of DNA barcoding – a species identification system reliant on a short but standardized gene region (Hebert et al. 2003a,b). Although species identification is the primary role for this system, it has also been touted as a tool that can expedite the species discovery process by identifying genetically divergent populations within recognized species (Hebert et al. 2004a,b).

How much genetic divergence is necessary to distinguish species is a question that has incited controversy (Moritz and Cicero 2004; DeSalle et al. 2005; Meyer and Paulay 2005; Wiemers and Fiedler 2007). Proponents have advocated various metrics, most notoriously the “10x rule” (Hebert et al. 2004b). This and other thresholds have spurred critics who contend that a threshold of genetic divergence is not an accurate reflection of the speciation process (Hickerson et al. 2006). Kerr et al. (2009a) found that a multi-tiered approach, incorporating additional methods such as character-based measures, was the most effective in accurately portraying species limits, but this approach is heavily reliant on a priori knowledge of species boundaries and performs poorly as a species discovery tool. The current consensus is that a surveying method such as DNA barcoding is best implemented as a first pass approach and that such data should be complemented by alternative sources of evidence before taxonomic boundaries are reconsidered (DeSalle 2006; Padial and de la Riva 2007; Frezal and Leblois 2008).

DNA barcode coverage of extant birds is more comprehensive than in any other taxonomic group. This is attributable to several large-scale geographically oriented surveys (Yoo et al. 2006; Kerr et al. 2007, 2009a,b; Johnsen et al. 2010) and, more recently, to the systematic sampling of one of the world's largest avian museum collections (Schindel et al. 2011). These studies invariably highlight species harboring large intraspecific divergences. Many of these species have already been the subject of more rigorous analyses and simply await authoritative decisions (e.g., Barker et al. 2008; Areta and Pearman 2009), while divergences within other species remain poorly studied, or unknown, and require further review. The Northern Fulmar (*Fulmarus glacialis*) is a good example from the latter category.

The Northern Fulmar is a widespread seabird in the northern hemisphere, though Atlantic and Pacific populations occur disjunctively (Hatch and Nettleship 1998). Generally, three subspecies are recognized (*sensu* Clements 2007), one in the Pacific (*F. g. rodgersii*) and two in the Atlantic (the smaller-billed, high arctic breeder *F. g. glacialis*, and the larger-billed, boreal *F. g. auduboni*). Northern Fulmars display highly variable plumage, exhibiting light and dark morphs plus a large suite of intermediates, which historically has helped confound taxonomic boundaries. For example, pale-morph birds from the north Pacific were initially described as a separate species, *F. rodgersii* (American Ornithologists' Union 1910), while the remainder of Pacific birds were then known as *F. g. glupischa* (Bent 1964). An additional subspecies was also initially described from the Canadian arctic (*F. g. minor*), but was later synonymized with the nominate subspecies; some authors have prescribed the same treatment for *F. g. auduboni* (van Franeker and Luttik 2008). Currently, the American Ornithologists' Union (1998) recognizes *F. glacialis* as a single species.

Although the two color morphs occur in both the Atlantic and Pacific, their degree of contrast and their latitudinal frequencies vary substantially between these populations. Both the palest and the darkest birds occur in the Pacific, but intermediates are rare, whereas in the Atlantic light and dark morphs contrast less and intermediates are more common (Hatch and Nettleship 1998; Sibley 2000). An even more curious pattern is that light birds in the Pacific tend to be concentrated at higher latitudes, whereas the reverse trend is observed in the Atlantic (van Franeker and Wattel 1982; Warham 1990). To explain this unusual pattern, van Franeker and Wattel (1982) postulated a Pacific origin for the dark morphed birds and suggested that dark Pacific birds could have subsequently invaded the Atlantic during warm interglacial periods, and light Atlantic birds could have contemporaneously recolonized the north Pacific. Under this hypothesis, the authors predicted that additional characters should unite birds by color morph rather than by geography, but this hypothesis has not been formally tested.

While the life history and general biology of the Northern Fulmar has been studied in depth (e.g., Fisher 1952), genetic data is largely absent for this species (Hatch and Nettleship 1998). DNA barcode surveys based on cytochrome *c* oxidase I (COI) included only a single individual from the Pacific Ocean, but it was found to be roughly 3.2% divergent (based on a Kimura 2-parameter distance model) from the remainder of specimens analyzed, which all originated from sites distributed throughout the North Atlantic (Kerr et al. 2007; Johnsen et al. 2010). However, the color morph of the specimens was not reported in these studies.

In this study, we examine sequence data from both nuclear and mitochondrial loci to obtain a better grasp of genetic variation within the Northern Fulmar. We use multilocus phylogeographic analyses to assess genetic divergence between the Atlantic and Pacific populations and test the hypothesis that dark morph birds evolved in the Pacific. We include in our genetic survey a fragment from the melanocortin-1 receptor gene, from which mutations have been associated with melanic plumage in a host of other species including many avian examples (Mundy 2005). The melanocortin-1 receptor is a G protein-coupled receptor that affects the activity of melanocytes. It is believed that some amino acid substitutions can alter its activity resulting in constitutive activation, effectively leaving the receptor ‘on’, and resulting in an overproduction of eumelanin (Ling et al. 2003). We determine if mutations in this gene are correlated with dark plumage in the Northern Fulmar.

## Methods

### Taxon sampling

**Figure 1:**
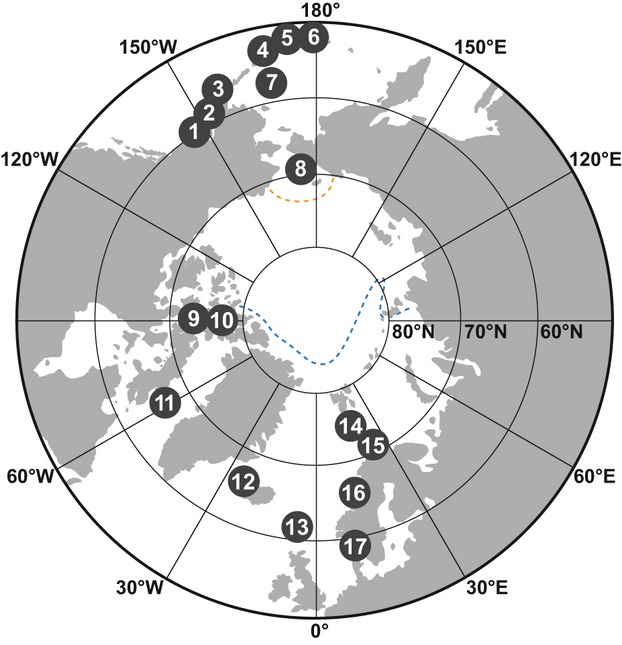
Specimens analyzed in this study originated from 17 collecting sites: 1) Kenai Peninsula, US; 2) Gulf of Alaska; 3) Chowiet I., US; 4) Chagulak I., US; 5) Adak I., US; 6) Amchitka I., US; 7) St. George I., US; 8) Chukchi Sea; 9) Prince Leopold I., CA; 10) Devon I., CA; 11) Baffin I., CA; 12) Faxaflói, IS; 13) Faeroe I., DK; 14) Bear Island, NO; 15) Nordkapp, NO; 16) Trænabanken, NO; 17) Tromlingene, NO. Dashed lines represent the northern range limits for Atlantic (blue) and Pacific (orange) populations, respectively.

Tissue samples were obtained from various frozen tissue collections and were composed of pectoral muscle or liver samples (see Table S1 for complete list of specimens). One hundred and thirty-four Northern Fulmars from 17 different locations were sampled in total (Fig. 1); 81 samples represented Atlantic populations and 53 were from the Pacific. Whenever possible, tissues with associated voucher skins were used so that color morph could later be determined. Two specimens of *Fulmarus glacialoides* were included in the analysis, plus single representatives from four of the five remaining members of the Fulmarine clade of petrels: *Macronectes giganteus*, *M. halli*, *Thalassoica antarctica*, and *Pagodroma nivea*.

### DNA extraction, amplification, and sequencing

DNA was extracted from all tissue samples using the DNeasy blood and tissue kit (Qiagen, Valencia, CA), following the manufacturers’ instructions. DNA samples were eluted in 150 μL of ultrapure water. All polymerase chain reactions (PCR) were performed in total volumes of 12.5 μL (8.25 μL molecular grade H_2_O, 1.25 μL 5× buffer, 0.625 μL 25 mM MgCl_2_, 0.125 μL of each primer (10 mM), 0.0625 μL 2.5 mM each dNTPs, 0.06 μL GoTaq® Flexi polymerase (Promega, Madison, WI), and 2 μL DNA template). For difficult samples, Platinum® Taq polymerase (Invitrogen, Carlsbad, CA) was used as a substitute along with the manufacturer's reagents. All primer sequences and citations are listed in Appendix 1.

A 694-bp fragment of the mitochondrial gene cytochrome *c* oxidase I (COI) was amplified using the primer pair BirdF1/COIbirdR2 and the following thermal cycle: 94°C for 2 min; 30 cycles of 95°C for 30 sec, 48°C for 40 sec, 72°C for 45 sec; 72°C for 5 min. Alternative primer pairs included BirdF1/AvMiF1 and COIbirdR2/AvMiR1, which targeted smaller amplicons using internal primers, and LTyr/COI907aH2, which amplified a larger amplicon including part of the tyrosine tRNA.

A 298 bp fragment of the mitochondrial control region (CR) was amplified using the primer pair ND6*-L16406/H505 and the same thermal cycle described above. A 691 bp fragment of myoglobin intron II (MYOII) was amplified using half-nested PCR with the primers Myo2/Myo3 for the initial reaction and Myo2/Myo3F for the second. Both thermal cycles matched that described above, except with an annealing temperature of 59°C rather than 48°C. An 817 bp fragment of melanocortin-1 receptor (MC1R) was amplified using the primer pair MSHR9/MSHR72 and the thermal cycle described above, except with an annealing temperature of 61°C rather than 48°C. Additionally, two pairs of internal primers were designed to amplify sequences from more challenging samples: MC1RNoFu63/MC1RNoFu532 and MC1RNoFu362/MC1RNoFu802. A 499 bp fragment of “intron A” of the Z-linked chromodomain-helicase DNA-binding protein 1 (CHD1-Z) gene was amplified using the primer pair 2669FZ/2718R and the following touchdown thermal cycle: 94°C for 2 min; 10 cycles of 94°C for 30 sec, 60°C (decreasing by 1°C/cycle) for 30 sec, 72°C for 40 sec; 30 cycles of 94°C for 30 sec; 50°C for 30 sec, 72°C for 40 sec; 72°C for 5 min.

PCR templates were purified using ExoSAP-IT® (USB corporation, Cleveland, OH) prior to cycle sequencing. All sequencing reactions used the same primers as for PCR, except for LTyr and COI907aH2, which were sequenced using COIaRt and COI748Ht, respectively. Sequencing was performed bi-directionally (except for CR, which was sequenced with only H505) on an ABI 3730xl sequencer (Applied Biosystems, Foster City, CA). All sequences produced for this study were submitted to GenBank (accession numbers KC755410-KC755549, COI; KC755550-KC755682, CR; KC788940-KC789071, MYOII; KC755695-KC755826, MC1R; KC788823-KC788939, CHD1-Z), as well as uploaded to the data project “Fulmarine Petrels” in BOLD (http://www.boldsystems.org).

### Phylogenetic analysis

Alignments were made by eye for each gene fragment using BioEdit version 7.0.5.3 (Hall 1999). Sequences were trimmed to equal lengths where necessary. For nuclear sequences containing heterozygous peaks, the allelic phase of each haplotype was resolved via one of two methods: i) simple heterozygous sequences – that is, with only one or two point mutations – were resolved using the SNP data file format in PHASE version 2.1 (Stephens et al. 2001); ii) complex heterozygous sequences were resolved by cloning. Cloning was performed using the TOPO TA Cloning kit (Invitrogen). To accommodate heterozygosity, secondary alignments were produced for nuclear gene fragments wherein each specimen was represented by two sequences, one to represent each copy (this was not done for CHD1-Z).

For each marker, we calculated haplotype diversity, nucleotide diversity, Tajima's D, and Fu & Li's D* and F* statistics using DnaSP version 5.0 (Rozas and Librado 2009), first using the entire population, and then measuring the Atlantic and Pacific populations separately. Median-joining haplotype networks were also developed separately for each marker using Network 4.6.0.0 (http://www.fluxus-engineering.com).

Phylogenetic trees were constructed using concatenated datasets. Separate trees were built for nuclear and mitochondrial markers; however, CHD1-Z was omitted from the nuclear concatenated data file due to lack of variation. For each concatenated dataset, the best-fit evolutionary model and partition scheme were selected using the Bayesian information criterion (BIC) in the program PartitionFinder version 1.0.1 (Lanfear et al. 2012). Model selection was limited to those implemented in MrBayes version 3.1.2. For each dataset, Bayesian phylogenetic trees were constructed with MrBayes version 3.1 (Huelsenbeck and Ronquist 2001; Ronquist and Huelsenbeck 2003). Data partitions are described in the results. For both datasets all parameters were unlinked, the sampling frequency was set to 5,000, and the first 25% of samples were discarded as burn-in. Each of the analyses were run sufficiently long for the average standard deviation of split frequencies to fall below 0.01 (25,000,000 generations for the nuclear dataset and 10,000,000 for the mitochondrial dataset). The initial results of the nuclear dataset yielded unusually long branch lengths, which is a well-documented ailment of partitioned datasets in MrBayes (Marshall 2010). To solve this problem, we repeated the analysis for the nuclear dataset with the branch length prior mean reduced to 1/100 as recommended by Ward et al. (2010). *Pagodroma nivea* was selected as the outgroup taxa for both trees (*sensu* Penhallurick and Wink 2004).

### Estimate of divergence time

Divergence time was only estimated for the mitochondrial lineages (see Discussion) and was based on COI using the program BEAST v1.7.1 (Drummond et al. 2012). Each population of *F. glacialis* was represented by a single sequence. COI sequences from additional species were downloaded from BOLD to provide reasonable coverage of all Procellariiform families, plus an outgroup from the Sphenisciformes; 27 taxa were included in total (Appendix 2). Because variable molecular rates have been proposed for the Procellariiformes (e.g., Nunn and Stanley 1998; Weir and Schluter 2008) we instead used an uncorrelated lognormal relaxed molecular clock with a Yule tree prior and three fossil calibration points to date the tree: *Thalassarche-Phoebetria*, 5.3 Mya (Wilkinson 1969); Hydrobatidae, 10.25 Mya (Becker 1987); and Diomedeidae 30–31 Mya (Mayr and Smith 2012). All calibration dates used exponential priors with means set to 1.0 (Ho 2007). The GTR+Γ substitution model was selected based on jModeltest (Posada 2008). The gamma shape parameter and substitution parameter priors were also informed by the jModeltest results. For the clock mean, a broad gamma distribution (shape 0.001, scale 1000) was used. Default prior values were accepted for the remaining parameters. The program was run for 50 million generations and sampled every 5,000 generations. The program was rerun subsequently sampling only from the prior to observe the influence on the results. Tracer v1.5 (http://tree.bio.ed.ac.uk/software/tracer/) was used to assess convergence and assure that all effective sample sizes (ESSs) were at least greater than 200.

### MC1R and color morphs

Northern Fulmar color morphs are typically divided into four categories: double light, light, dark, and double dark (van Franeker and Wattel 1982; Hatch 1991). For the purpose of this study, only three categories were necessary (two ‘pure bred’ categories, plus hybrids), so birds were only distinguished as being light (= double light), dark (= double dark), or intermediate (= light and dark categories).

Aligned MC1R sequences were translated using the standard genetic code. A previous prediction of the two-dimensional structure of MC1R in birds as collated by Mundy (2005) was used to map amino acid substitutions in the Northern Fulmar to transmembrane and loop sites. Variable amino acid positions were inspected individually for possible correlations between substituted amino acids and dark plumage (light plumage is presumed to be the ancestral condition on the basis of its absence in the more ancestral southern species), and were compared to substitutions linked to melanism in other species.

## Results

### Sequencing results

Not all markers could be amplified from every specimen (see Appendix 3 or Table S1). Additionally, some ambiguous base calls could not be resolved from the 3′ end of several CR and MYOII sequences, so the alignments for these two genes were trimmed to 228 bp and 650 bp, respectively, to eliminate the affected regions.

Heterozygous peaks occurred in 35% of MYOII sequences and 28% of MC1R sequences. In MYOII, peaks were primarily limited to single occurrences within any given sequence (two at most) and were easily resolved by phasing. In contrast, heterozygous peaks in MC1R numbered as high as eight in several sequences, often appeared at associated sites, and frequently resulted in amino acid substitutions. Twenty-one MC1R sequences could be resolved by phasing, but the remaining 14 necessitated cloning. No heterozygous peaks were observed in CHD1-Z but variation was generally low and any female specimens would only carry a single copy of this gene.

### Phylogenetic results

**Figure 2:**
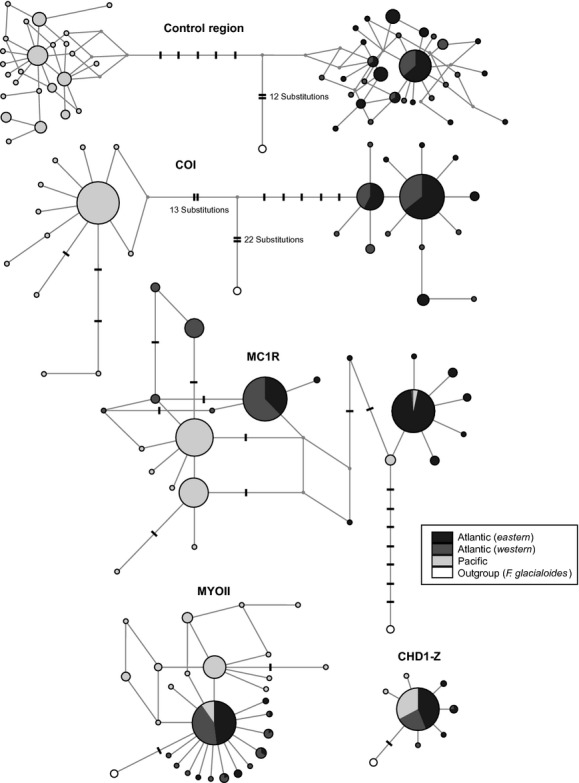
Median-joining haplotype networks for each of the five markers included in this study. The size of the circle is proportional to the number of individuals (CR, COI, and CHD1-Z) or alleles (MC1R and MYOII) with that haplotype. Adjoining circles are separated by a single nucleotide substitution. Additional substitutions are indicated by either hatch marks or, for larger values, a double hatch mark and a number. Dots indicate an unobserved median vector. Shading indicates the specimen's region of origin (the Atlantic region is divided into areas east and west of Greenland, respectively).

Twelve COI haplotypes were recovered from 53 Pacific birds, and 14 COI haplotypes were recovered from 81 Atlantic birds (Fig. 2). Variation was highly conserved in the Pacific, with 79% of individuals sharing the most common haplotype. The distribution in the Atlantic was more bimodal with 56% of individuals sharing the most common haplotype and 23% sharing the second most common; however, this split did not correlate to any geographical distribution (Fig. 2). CR haplotypes were more variable, with 25 haplotypes recovered from the Pacific and 28 from the Atlantic. Although, in the Atlantic 40% of birds still shared the most common haplotype. Both COI and CR clearly differentiated the Atlantic and Pacific populations with no haplotype sharing. For both markers, the Pacific population exhibited greater divergence from the outgroup taxa.

Haplotype sharing was observed for each of the nuclear markers, though to varying degrees (Fig. 2). The most extreme case was CHD1-Z, in which 91% of all birds shared the most common haplotype. MYOII presented a more typical case, where a subset of Pacific birds retained the most common haplotype from the Atlantic. MC1R displayed a strongly bimodal distribution within each population and also fewer rare haplotypes. In the Atlantic, one of the common haplotypes was predominantly restricted to areas east of Greenland (Fig. 2). This is also the only MC1R haplotype shared with a Pacific bird, although it was limited to one individual (this sample was reextracted and resequenced for confirmation). A very similar haplotype was recovered from birds of the Pribilof Islands (site #7 in Fig. 1), but was only ever observed in single copy. This haplotype excepted, MC1R and MYOII showed greater divergence in the Pacific as the mitochondrial markers did.

**Figure 3:**
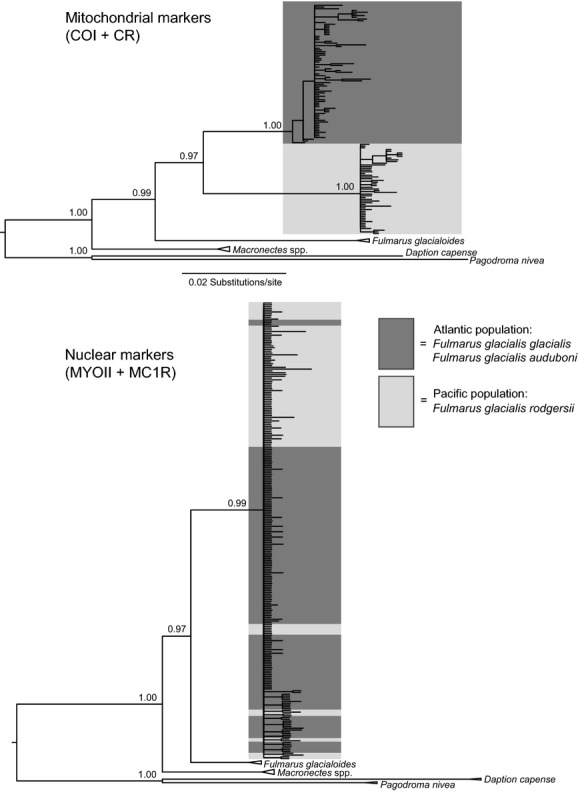
Concatenated gene trees based on mitochondrial and nuclear markers, respectively, estimated using MrBayes. The nuclear tree is based on phased haplotypes.

The concatenated mitochondrial gene tree was cleanly bifurcated into Atlantic and Pacific clades with high support (Fig. 3). As indicated by the network diagrams, the Atlantic clade showed no further highly supported subdivisions. The concatenated nuclear gene tree did not differentiate the Atlantic and Pacific clades, but grouped them into one highly supported cluster (Fig. 3). This is presumably due to haplotype sharing, and hence the haplotype networks offer a more informative depiction of the variation observed via nuclear markers.

### Divergence time

All ESS values greatly exceeded the minimum required (mean = 4054.11, range = 465.68–9001); however, the 95% highest posterior densities (HPD) for most node ages were relatively broad. The divergence time for the Atlantic and Pacific Northern Fulmar populations was estimated at 1.97 Ma (HPD = 0.72−3.37). Divergence between the Northern and Southern Fulmars was estimated at 2.95 Ma (HPD = 1.42−4.66), and between *Fulmarus* and *Macronectes* at 4.56 Ma (HPD = 2.42−6.76).

### MC1R and color morphs

**Figure 4:**
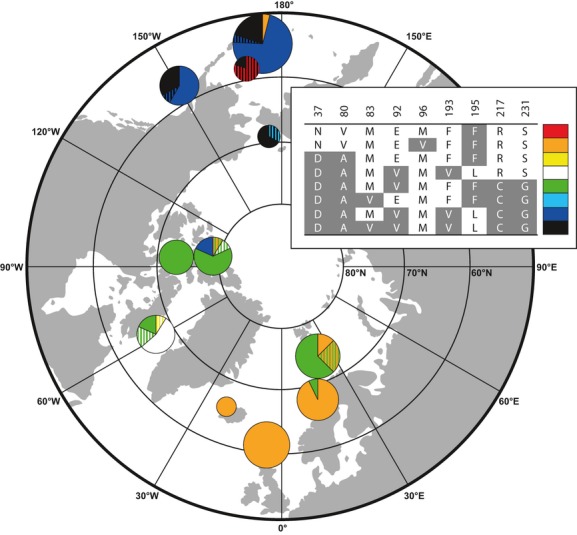
Geographical distribution of the eight most common MC1R amino acid haplotypes. The amino acid at each relevant position in the sequence is depicted in the inset for each haplotype, which are then coded by color. Circles on the map are proportional to the number of individuals represented at each site. Whole colors represent homozygous individuals and hatched colors represent the composition of heterozygous individuals.

Of 134 specimens with MC1R sequences, 79 had associated voucher skins. The bulk of specimens lacking this information were from the Canadian arctic islands, from which no specimens included vouchers. In the MC1R fragment sequenced in this study, excluding a few low frequency mutations (i.e., those occurring in only one or two individuals) there were nine notable sites where amino acid polymorphisms were found, a couple of which were linked pairs (Fig. 4). At least one of these mutations, E92V, occurs at a site implicated in melanism in other species (Mundy 2005).

**Figure 5:**
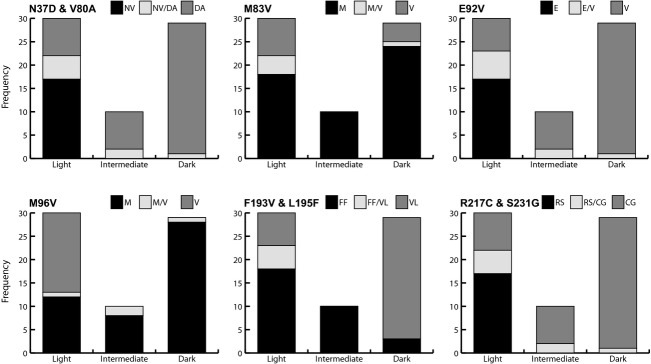
Frequency of amino acid composition at six individual or linked sites in the MC1R amino acid sequence. Shading indicates whether individuals were homozygous (black or dark gray) or heterozygous (light gray). Composition frequency is given for each plumage category: light, dark, and intermediate.

Despite the relatively large number of polymorphic sites, none of these consistently correlated to color morph throughout the species range (Fig. 5). Some weak patterns did emerge. For example, all birds homozygous for glutamic acid at position 92 (the ancestral state) were light morph birds; however, in the Atlantic several birds homozygous for valine at this position had intermediate plumage, and in the Pacific some valine homozygotes were complete light morphs. The distribution of MC1R haplotypes appeared to be more heavily influenced by geography (Fig. 4). For example, some light morph birds from the Pacific differ at all of the nine polymorphic sites from light morph birds from the Atlantic.

## Discussion

### Genetic divergence in the Northern Fulmar

Both mitochondrial markers indicated strong divergence between Atlantic and Pacific populations of Northern Fulmar, supporting the pattern initially suggested by DNA barcoding results (Kerr et al. 2007; Johnsen et al. 2010) but differing from the current taxonomic recognition (American Ornithologists' Union 1998). However, not even the fast-evolving control region indicated any clear divergence *within* the Atlantic population, thus failing to support the presence of two separate evolutionary significant units in that region. Results from nuclear markers were less clear. Haplotype sharing occurred in each of the three markers but to varying degrees. Sharing was most prominent for CHD1-Z, which featured almost no variation at all. Consequently, haplotype networks were much more informative than the concatenated nuclear tree. The networks demonstrate that rare haplotypes were not shared between the Atlantic and Pacific population, suggestive of early-stage divergence.

The lack of diversity at the CHD1-Z locus is slightly unusual. The Z chromosome has a smaller effective population size than its autosomal counterparts on account of its single-copy occurrence in females. Additionally, sexual selection is also thought to result in faster divergence of Z-linked genes (Borge et al. 2005). The low diversity observed within and between populations might seem to implicate male-biased gene flow and question the integrity of any postzygotic boundaries between the populations since the Z chromosome has recently been regarded as a “hotspot” for speciation genes (Sæther et al. 2007). However, CHD1-Z did not just demonstrate low variation between populations but also between other Fulmarine species. The CHD1-Z sequence in the focal taxa only differed from its congener, *F. glacialoides*, by two substitutions. Additionally, *F. glacialoides* sequences only differed from those of the genus *Macronectes* by a single substitution, and the two *Macronectes* species showed no interspecific variation at all. Further to this point, female Procellariiformes are known to disperse more than males (Burg et al. 2003). These results suggest “intron A” of the Z chromosome may simply be largely conserved in the Fulmarini clade.

In general, the discrepancy between the mitochondrial and nuclear markers is not surprising and is consistent with many similar studies (Welch et al. 2011; Gangloff et al. 2013). Time to reciprocal monophyly is generally correlated with generation time and effective population size (Avise et al. 1984; Hudson and Coyne 2002). Both of these measures are relatively large in the Northern Fulmar, with generation time generally estimated around 20 years (Koons et al. 2006; Jones et al. 2008) and census estimates suggesting both populations include millions of breeding pairs (Brooke 2004). These values collectively would predict a disproportionately long time to reciprocal monophyly relative to the divergence data, even for mitochondrial markers, though it should be borne in mind that modern population estimates might not be reflective of population trends at the time of divergence. Using values from Table 1 in Hudson and Coyne (2002) and our own divergence date estimates, it can be inferred that the long-term effective population size that would allow the observed mitochondrial divergence between Atlantic and Pacific birds would range from roughly 17,000 to 75,000 individuals/population. In turn, even the low end of this range would potentially require 2.9 million years to observe with high probability the reciprocal monophyly of a nuclear marker. This is consistent with our data, where nuclear markers appear to display reciprocal monophyly between *F. glacialis* and *F. glacialoides*, which our data suggests diverged ∼3 Mya.

The above estimates depend on the accuracy of the generation time estimates for Northern Fulmars. For example, if generation time were in fact closer to the average age at first reproduction (8–12 years, Hatch and Nettleship 1998) then the above population size estimates would double, but the values would still remain much below the current census population estimates. The settlement of the northern hemisphere is hypothesized to have followed a founder event from a southern ancestor traveling up the Pacific coastline (Voous 1949), which would likely result in a smaller initial population size in the north and subsequently a shorter predicted time to reciprocal monophyly. The division between the Atlantic and Pacific colonies on the other hand is more likely to have followed a vicariant event (i.e., the extension of Arctic ice), which theoretically would cleave the northern species into two more evenly sized populations. This too would help explain why monophyly is observed between *F. glacialis* and *F. glacialoides*, but not between *F. glacialis* populations. Detracting from this logic is the rapid population growth observed in the North Atlantic, where newly colonized locations such as the Faeroe Islands have seen populations surpass 500,000 breeding pairs in under 200 years (Fisher 1952). Alternatively, selective sweeps might also help explain the degree of mitochondrial divergence in *F. glacialis*, but evidence for such events is still largely lacking (Kerr 2011). The smaller effective population size of the mitochondrial genome appears to remain the most parsimonious explanation for the mito-nuclear discrepancy.

Northern Fulmars in the Pacific tend toward smaller body size and slimmer bills, whereas Atlantic birds are larger on average and bill size varies clinally (being smaller in the north), but measurements for both features overlap between these two regions (Brooke 2004; Pyle 2008; Howell 2012). Morphological differences are difficult to encapsulate due to the variable plumage, but Pacific birds are noted for having a darker tail that contrasts against a paler rump, whereas Atlantic birds are more uniformly colored across the tail and rump (Sibley 2000). Unfortunately, this feature is largely obscured or completely lost in Pacific dark morphs. Avian taxonomic decisions are often skewed toward characters that are diagnosable in the field, but this sometimes obscures distinct evolutionary lineages (Watson 2005).

One of the inherent challenges of studying divergent populations in allopatry is the inability to test the criteria of the biological species concept under natural conditions, i.e., assortative mating in sympatry (Mayr 1963). A common approach to taxonomic decisions for allopatric populations is to make comparisons to closely allied species pairs that do occur in sympatry (Helbig et al. 2002). Some authors have suggested that differences between Northern Fulmars from the Atlantic and Pacific are on par with that observed between other Procellariid species (Howell 2012). A notable example is *Macronectes giganteus* and *M. halli*, which differ only subtly by primarily qualitative morphological characters and exhibit far less genetic differentiation than the Northern Fulmar populations (Techow et al. 2010). Other examples, such as *Pterodroma phaeopygia* and *P. sandwichensis* echo the case of *Macronectes*, but these species breed on separate islands and may not be considered sympatric per se (Welch et al. 2011). It is hypothesized that high natal philopatry reduces selection for plumage divergence in Procellariids, which makes species recognition within this group very challenging in general (Brooke 2004).

The Northern Fulmar shares a common phylogeographic distribution with a few other seabird species and allospecies pairs, for example, *Uria aalge* (Morris-Pocock et al. 2008), *U. lomvia* (Birt-Friesen et al. 1992), and *Fratercula arctica/corniculata* (Friesen et al. 1996). Interestingly, divergence dates for these others pairs are variable, with the youngest only being 56,000–226,000 years ago (Morris-Pocock et al. 2008). Land and ice are common barriers to dispersal for all of these species (Friesen et al. 2007), so it is curious that they don't share a common divergence date. These dates are primarily predicted based on molecular clock estimates, so it could be that rate heterogeneity is underappreciated. However, the Northern Fulmar's exceptional generation time would predict a slower evolutionary rate compared to the other species, yet it yields the oldest divergence date for the group. If the other species were able to cross the Arctic passage at later dates, it would be reasonable to surmise that there would be no barrier to prevent the Northern Fulmar from completing similar movement, but the integrity of the populations remains intact.

Two historical features remain at odds with our findings: the *Fulmarus* fossil record and the Pacific coast origin hypothesis. Two prehistoric fossils from Kern County, California are attributed to the genus, including the proximal end of a carpometacarpus identified as *F. hammeri* (Howard 1969) and a complete humerus identified as *F. miocaenus* (Howard 1984). These fossils were said to belong to the Barstovian and Clarendonian stages, respectively, which roughly range from 10 to 16 Mya. This timeline vastly predates our estimates for the time to most recent common ancestor for even *Fulmarus-Macronectes*, and instead is more in line with the origin of the entire Fulmarini clade. Given that we used well-accepted fossil calibrations to obtain our date estimates, we propose that the Howard fossils likely belonged to either an ancestral lineage or to independent lineages with no modern descendents, but in either case should not be included in the genus *Fulmarus*. Wetmore (1926) describes a fossilized fragment of a left humerus collected in Maryland that is so similar to the modern day species that it “cannot be distinguished from [Fulmarus] glacialis”, but while he speculated that this fossil dated back to the Miocene (on account of its dark coloration and degree of fossilization), he also stated that the stratum from which it was collected is more consistent with a Pleistocene origin. The latter scenario would be consistent with our data.

Voous (1949) proposed that the Northern Hemisphere was colonized by ancestral fulmars traveling up the cooler water of the west coast of the Americas. He used bill shape to support this hypothesis, indicating that the slender bill of *F. glacialoides* was the ancestral condition and the broader bill of the North Atlantic *F. glacialis* was the most derived (the relatively slim bill of Pacific birds would thus reflect an intermediate state). This hypothesis has perpetuated unchallenged (Hatch and Nettleship 1998), though before Voous (1949) it was thought that the Atlantic was colonized *prior* to the Pacific (Fisher and Waterston 1941). Contrary to Voous' hypothesis, our molecular data reveal more derived characters in the sequences of Pacific birds for four out of five markers (CHD1-Z was the exception because the two populations predominantly shared a single haplotype). This could detract from the Pacific coast origin hypothesis, though it is possible that the increased divergence in the Pacific is reflective of that region's smaller population size, which can have a positive effect on the molecular evolutionary rate (Ohta 1973).

### MC1R and color morphs in the Northern Fulmar

The association between MC1R mutants and melanism was first deduced in model laboratory species such as mouse and subsequently chicken, but a common role was later confirmed in such disparate avian taxa as skuas, geese, and passerines (see Mundy 2005 for review). MC1R has since been regularly employed as a candidate gene in avian plumage studies, though often arguably in vain. Although MC1R-melanism correlations have been found in additional avian taxa (Baiao et al. 2007; Pointer and Mundy 2008; Uy et al. 2009; Vidal et al. 2010), a near equal number of studies have unsurprisingly yielded negative results (MacDougall-Shackleton et al. 2003; Cheviron et al. 2006; Haas et al. 2009; Hull et al. 2010; Cadena et al. 2011; Dobson et al. 2012). Several of the latter studies examined species that exhibited finely scaled patterns, not melanism per se (MacDougall-Shackleton et al. 2003; Cadena et al. 2011; Dobson et al. 2012). Others examined species where melanic plumage was limited to a single sex and age class (Cheviron et al. 2006).

In the absence of a functional analysis, there is an intrinsic risk of falsely attributing cause to MC1R mutations when it could simply correlate with plumage variation because of population divergence. Doucet et al. (2004) described an association between MC1R and melanism in the island subspecies of *Malurus leucopterus*, which features melanic plumage in males, but Mundy (2005) correctly speculated that this association might be an artifact of population divergence, as was later demonstrated by Driskell et al. (2010). In his review, Mundy (2005) offers strong evidence for how demographic history can be ruled out as an explanation for the association in at least three iconic species, which bolsters the role of MC1R in at least some cases. Interestingly, shared amino acid substitutions are often recorded in unrelated species (Mundy 2005), which seemingly gives further credence to causation, but these substitutions are also found in nonmelanic species (Pointer and Mundy 2008). If MC1R mutations do in fact play a role in determining color morph in the Northern Fulmar then a strenuous explanation would be required to explain the mechanism.

## Conclusions

The distribution and degree of variation of color morphs across the range of *F. glacialis* has long been of interest. Determining the genetic cause of this variation will prove to be equally interesting, but might remain a challenge for some time. The gene implicated in melanic plumage in several other avian examples, MC1R, shows a surprising amount of nonsynonymous variation within *F. glacialis*, yet this variation does not correlate with the plumage color of specimens.

The current taxonomic status of *F. glacialis* is inconsistent with the treatment of other species within the Procellariiformes. The Atlantic and Pacific populations display genetic (and morphological) divergence comparable to that observed between other Procellariid sister species, and appear to be on independent evolutionary trajectories. Divergence date estimates indicate that the Atlantic and Pacific populations may have persisted independently for nearly 2 million years, spanning over warm interglacial periods that may have drastically reduced ice cover (Anderson et al. 2006). Consequently, taxonomic boundaries within this genus warrant reconsideration and we would recommend elevating the Pacific population to a separate species. Despite the significant genetic divergence between these two populations, not even fast-evolving regions such as the mitochondrial control region distinguish the two named Atlantic subspecies and thus we support the decision of some authors not to recognize *F. g. auduboni* as a valid subspecies.

The association of genetic markers and geographical distribution but not color morph fails to support an old hypothesis on the origin of the Northern Fulmar's modern day color morph distribution (van Franeker and Wattel 1982). The Northern Fulmar is just one of several seabirds with ranges bisected by Arctic ice. Some current climate predictions indicate that summer arctic sea ice is likely to recede within the lifetime of some contemporary biologists (Kerr 2009), which may present a unique opportunity for future biographical study if long separated populations are reunited.

## Data Archive

DNA sequences have been deposited in GenBank (accession numbers: KC755410-KC755682, KC755695-KC755826, KC788823-KC789071) and BOLD (in the project FULMR Fulmarine Petrels). Alignments of phased DNA sequences for MYOII and MC1R have been deposited in the Dryad Repository (doi:10.5061/dryad.0520f).

## Appendix 1

Sequences and citations for primers used in this study.

**Table:**

Primer	Sequence (5′ to 3′)	Gene	Dir.	Citation
BirdF1	TTCTCCAACCACAAAGACATTGGCAC	COI	F	(Hebert et al. 2004b)
COIbirdR2	ACGTGGGAGATAATTCCAAATCCTGG	COI	R	(Hebert et al. 2004b)
AvMiF1	CCCCCGACATAGCATTCC	COI	F	(Kerr et al. 2009b)
AvMiR1	ACTGAAGCTCCGGCATGGGC	COI	R	(Kerr et al. 2009b)
LTYR	TGTAAAAAGGWCTACAGCCTAACGC	COI	F	(Tavares and Baker 2008)
COI907aH2	GTRGCNGAYGTRAARTATGCTCG	COI	R	(Tavares and Baker 2008)
COIaRt	AACAAACCACAAAGATATCGG	COI	F	(Tavares and Baker 2008)
COI748Ht	CCAGGCTTYGGAATTATYTCCCA	COI	R	(Tavares and Baker 2008)
ND6*-L16406	CCACCCCATAATACGGCGAAGG	CR	F	(Burg et al. 2003)
H505	GAAAGAATGGTCCTGAAGC	CR	R	(Burg et al. 2003)
Myo2	GCCACCAAGCACAAGATCCC	MYOII	F	(Slade et al. 1993)
Myo3	CGGAAGAGCTCCAGGGCCTT	MYOII	R	(Slade et al. 1993)
Myo3F	TTCAGCAAGGACCTTGATAATGACTT	MYOII	R	(Heslewood et al. 1998)
MSHR72	ATGCCAGTGAGGGCAACCA	MC1R	F	(Mundy et al. 2004)
MSHR9	CTGGCTCCGGAAGGCATAGAT	MC1R	R	(Mundy et al. 2004)
MC1RNoFu63	GCAACCAGAGCAACACCAC	MC1R	F	*This study*
MC1RNoFu802	GAAGAAGCAGGTGCAGAAGG	MC1R	R	*This study*
MC1RNoFu362	GGACAACGTCATCAACATGC	MC1R	F	*This study*
MC1RNoFu532	GCGTTGTTGTGGTAGTAGGTGA	MC1R	R	*This study*
2669FZ	CTCAGAT GGTGAGGATGCTG	CHD1-Z	F	(Berlin et al. 2006)
2718R	ATTGAAATGATCCAGTGCTTG	CHD1-Z	R	(Fridolfsson and Ellegren 1999)

Run direction is indicated by F (forward) or R (reverse).

## Appendix 2

List of all COI sequences included in the BEAST divergence dating analysis.

**Table:**

BOLD Process ID	GenBank no.	Species	Family
FULMR104-10	KC755410	*Fulmarus glacialis* (Atlantic)	Procellariidae
FULMR116-11	KC755530	*F. glacialis* (Pacific)	Procellariidae
KPARG309-08	KC755545	*F. glacialoides*	Procellariidae
FULMR121-11	KC755546	*Macronectes giganteus*	Procellariidae
FULMR122-11	KC755547	*M. halli*	Procellariidae
BROMB704-07	n/a	*Daption capense*	Procellariidae
FULMR016-10	KC755549	*Thalassoica antarctica*	Procellariidae
FULMR120-11	KC755548	*Pagodroma nivea*	Procellariidae
GBIR1243-09	AY567883	*Calonectris diomedea*	Procellariidae
BROMB706-07	n/a	*Procellaria westlandica*	Procellariidae
ROMC325-07	n/a	*Pterodroma cookii*	Procellariidae
SIBHI087-11	JF498895	*Puffinus pacificus*	Procellariidae
KAARG101-07	FJ027984	*Pelecanoides georgicus*	Pelecanoididae
KAARG032-07	FJ027985	*P. magellani*	Pelecanoididae
ROMC300-07	n/a	*P. urinatrix*	Pelecanoididae
NZCOI449-09	n/a	*Phoebetria palpebrata*	Diomedeidae
NZCOI234-08	JW1033	*Diomedea epomophora*	Diomedeidae
BROMB337-06	n/a	*D. exulans*	Diomedeidae
SIBHI060-11	JF498886	*Phoebastria immutabilis*	Diomedeidae
KKBNA002-04	DQ433935	*P. nigripes*	Diomedeidae
KBARG059-07	FJ028397	*Thalassarche chlororhynchos*	Diomedeidae
KPARG374-08	n/a	*T. melanophris*	Diomedeidae
KPARG321-08	n/a	*Oceanites oceanicus*	Hydrobatidae
TZBNA330-03	AY666400	*Oceanodroma castro*	Hydrobatidae
TZBNA229-03	AY666306	*O. furcata*	Hydrobatidae
BON034-06	GU571506	*O. leucorhoa*	Hydrobatidae
KPARG109-08	n/a	*Spheniscus magellanicus*	Spheniscidae

## Appendix 3

Characterization of nucleotide polymorphism within and between populations, including number of segregating sites (S), number of haplotypes (H), nucleotide diversity (π), Watterson's estimator (θ_W_), and several neutrality tests calculated using DnaSP version 5.0 (Rozas and Librado 2009).

**Table:**

Population	n	S	H	π·10^−3^	θ_W_·10^−3^	Tajima's D	Fu & Li's D*	Fu & Li's F*
COI (694 bp)	134	35	24	0.51	9.52	1.6688	−1.4569	−0.21859
Atlantic	81	13	14	1.39	3.77	−1.7565	−3.0538*	−3.0844**
Pacific	53	9	10	0.56	2.95	−2.2444***	−3.9924**	−4.0287**
CR (228 bp)	131	42	62	40.52	33.81	0.1958	−2.3238	−1.5312
Atlantic	78	25	30	8.49	22.06	−1.9949*	−3.5059**	−3.5106**
Pacific	53	22	32	13.14	21.26	−1.2214	−1.4206	−1.6044
MYOII (694 bp)	252 (2·126)	21	27	1.37	5.74	−2.0075*	−4.3904**	−4.1363**
Atlantic	164 (2·82)	12	13	0.60	3.25	−2.0281*	−2.6307*	−2.8806*
Pacific	88 (2·44)	12	15	1.86	3.97	−1.4359	−2.2006	−2.2943
CHD1-Z (694 bp)	112	6	7	0.44	2.81	−1.8639*	−2.7728*	−2.9186*
Atlantic	75	4	5	0.52	2.03	−1.5573	−1.3637	−1.6707
Pacific	37	2	3	0.22	0.96	−1.4933	−2.3888	−2.4664
MC1R (694 bp)	252 (2·126)	21	23	6.09	4.73	0.7600	−1.8218	−0.9439
Atlantic	162 (2·81)	16	15	5.61	3.88	1.1732	−0.0888	0.4677
Pacific	90 (2·45)	15	8	2.17	4.06	−1.3083	−1.5951	−1.7762

**P* < 0.05; ***P* < 0.02; ****P* < 0.01.

## Supporting Information

**Table S1.** Complete list of all the specimens included in the analysis, their respective BOLD Process IDs, their respective GenBank accession numbers for each gene sequenced, their tissue composition, and the donor institution.
