# Corrigendum

**DOI:** 10.1002/ece3.1834

**Published:** 2015-11-17

Kevin C. R. Kerr & Carla J. Dove. Delimiting shades of gray: phylogeography of the Northern Fulmar, *Fulmarus glacialis*. Ecology and Evolution 2013; 3(7): 1915–1930. DOI: 10.1002/ece3.597

The branches labeled “*Daption capense*” in Figure 3 should have been labeled “*Thalassoica antarctica*”. The below figure has the correct label. This change has no impact on the overall conclusions of the paper. The authors regret this error.

**Figure 3:**
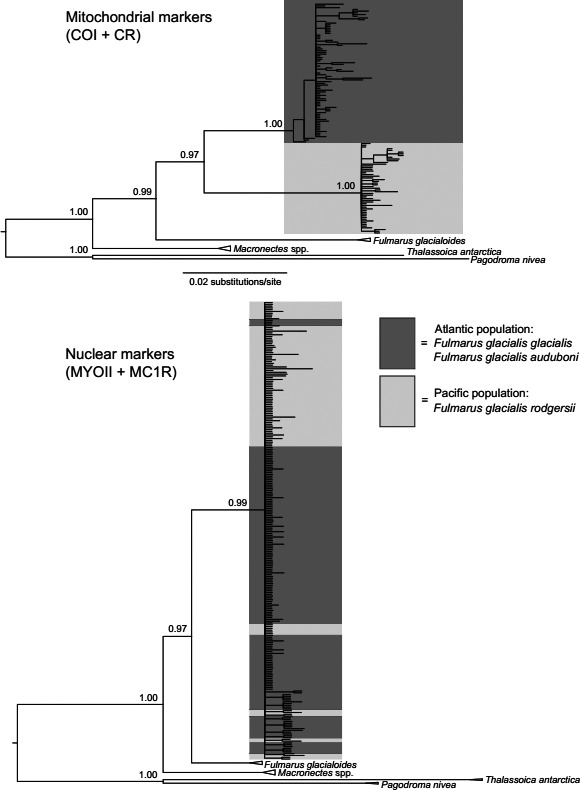
Concatenated gene trees based on mitochondrial and nuclear markers, respectively, estimated using MrBayes. The nuclear tree is based on phased haplotypes.

