# Supplementary material for: Delimiting shades of gray: phylogeography of the Northern Fulmar, Fulmarus glacialis
Source: Ecol Evol. 2013 May 22;3(7):1915–30. doi: 10.1002/ece3.597 (PMC3728934; doi:10.1002/ece3.597)
Supplement: Supplementary file 1 [file ece30003-1915-SD1.doc]

**Supplementary information:**

Table S1. Complete list of all the specimens included in the analysis, their respective BOLD Process IDs, their respective GenBank accession numbers for each gene sequenced, their tissue composition, and the donor institution.

| **Specimen** | **BOLD Process ID** | **Species** | **COI** | **CR** | **MYOII** | **MC1R** | **CHD1-Z** | **Tiss.*** | **Source†** |
| --- | --- | --- | --- | --- | --- | --- | --- | --- | --- |
| AMNH 1219 | FULMR017-10 | *F. glacialis* | KC755465 | KC755605 | n/a | n/a | n/a | M | AMNH |
| AMNH 1220 | FULMR018-10 | *F. glacialis* | KC755466 | KC755606 | n/a | n/a | n/a | M | AMNH |
| AMNH 1221 | FULMR019-10 | *F. glacialis* | KC755467 | KC755607 | n/a | n/a | n/a | M | AMNH |
| AMNH 1222 | FULMR020-10 | *F. glacialis* | KC755468 | KC755608 | n/a | KC755750 | n/a | M | AMNH |
| AMNH 1342 | FULMR025-10 | *F. glacialis* | KC755473 | KC755613 | KC788997 | n/a | n/a | M | AMNH |
| AMNH 1343 | FULMR026-10 | *F. glacialis* | KC755474 | KC755614 | n/a | n/a | n/a | M | AMNH |
| AMNH 1344 | FULMR021-10 | *F. glacialis* | KC755469 | KC755609 | KC788995 | KC755751 | n/a | M | AMNH |
| AMNH 1345 | FULMR022-10 | *F. glacialis* | KC755470 | KC755610 | n/a | n/a | n/a | M | AMNH |
| AMNH 1346 | FULMR023-10 | *F. glacialis* | KC755471 | KC755611 | KC788996 | KC755752 | n/a | M | AMNH |
| AMNH 1347 | FULMR024-10 | *F. glacialis* | KC755472 | KC755612 | n/a | KC755753 | n/a | M | AMNH |
| AMNH 1673 | FULMR027-10 | *F. glacialis* | KC755475 | KC755615 | n/a | KC755754 | n/a | M | AMNH |
| AMNH 1674 | FULMR028-10 | *F. glacialis* | KC755476 | KC755616 | KC788998 | n/a | n/a | M | AMNH |
| AMNH 1763 | FULMR029-10 | *F. glacialis* | KC755477 | KC755617 | n/a | n/a | n/a | M | AMNH |
| AMNH 1764 | FULMR030-10 | *F. glacialis* | KC755478 | KC755618 | KC788999 | KC755755 | n/a | M | AMNH |
| AMNH 1765 | FULMR031-10 | *F. glacialis* | KC755479 | KC755619 | KC789000 | KC755756 | n/a | M | AMNH |
| UAM 13011 | FULMR033-10 | *F. glacialis* | KC755411 | KC755551 | KC788941 | KC755696 | KC788824 | M | UAM |
| UAM 14072 | FULMR034-10 | *F. glacialis* | KC755412 | KC755552 | KC788942 | KC755697 | KC788825 | M | UAM |
| UAM 14073 | FULMR035-10 | *F. glacialis* | KC755413 | KC755553 | KC788943 | KC755698 | KC788826 | M | UAM |
| UAM 14074 | FULMR036-10 | *F. glacialis* | KC755414 | KC755554 | KC788944 | KC755699 | KC788827 | M | UAM |
| UAM 14075 | FULMR037-10 | *F. glacialis* | KC755415 | KC755555 | KC788945 | KC755700 | n/a | M | UAM |
| UAM 14076 | FULMR038-10 | *F. glacialis* | KC755416 | KC755556 | KC788946 | KC755701 | KC788828 | M | UAM |
| UAM 14077 | FULMR039-10 | *F. glacialis* | KC755417 | KC755557 | KC788947 | KC755702 | KC788829 | M | UAM |
| UAM 14078 | FULMR040-10 | *F. glacialis* | KC755418 | KC755558 | KC788948 | KC755703 | KC788830 | M | UAM |
| UAM 14079 | FULMR041-10 | *F. glacialis* | KC755419 | KC755559 | KC788949 | KC755704 | KC788831 | M | UAM |
| UAM 14080 | FULMR042-10 | *F. glacialis* | KC755420 | KC755560 | KC788950 | KC755705 | KC788832 | M | UAM |
| UAM 14081 | FULMR043-10 | *F. glacialis* | KC755421 | KC755561 | KC788951 | KC755706 | KC788833 | M | UAM |
| UAM 14082 | FULMR044-10 | *F. glacialis* | KC755422 | KC755562 | KC788952 | KC755707 | KC788834 | M | UAM |
| UAM 14083 | FULMR045-10 | *F. glacialis* | KC755423 | KC755563 | KC788953 | KC755708 | KC788835 | M | UAM |
| UAM 14084 | FULMR046-10 | *F. glacialis* | KC755424 | KC755564 | KC788954 | KC755709 | KC788836 | M | UAM |
| UAM 14085 | FULMR047-10 | *F. glacialis* | KC755425 | KC755565 | KC788955 | KC755710 | KC788837 | M | UAM |
| UAM 14086 | FULMR048-10 | *F. glacialis* | KC755426 | KC755566 | KC788956 | KC755711 | KC788838 | M | UAM |
| UAM 14087 | FULMR049-10 | *F. glacialis* | KC755427 | KC755567 | KC788957 | KC755712 | KC788839 | M | UAM |
| UAM 14088 | FULMR050-10 | *F. glacialis* | KC755428 | KC755568 | KC788958 | KC755713 | KC788840 | M | UAM |
| UAM 14105 | FULMR051-10 | *F. glacialis* | KC755429 | KC755569 | KC788959 | KC755714 | KC788841 | M | UAM |
| UWBM 72723 | FULMR008-10 | *F. glacialis* | KC755451 | KC755591 | KC788981 | KC755736 | KC788862 | M | UWBM |
| UWBM 72724 | FULMR009-10 | *F. glacialis* | KC755452 | KC755592 | KC788982 | KC755737 | KC788863 | M | UWBM |
| UWBM 72725 | FULMR010-10 | *F. glacialis* | KC755453 | KC755593 | KC788983 | KC755738 | KC788864 | M | UWBM |
| UWBM 78778 | FULMR011-10 | *F. glacialis* | KC755454 | KC755594 | KC788984 | KC755739 | KC788865 | M | UWBM |
| UWBM 78779 | FULMR032-10 | *F. glacialis* | KC755480 | KC755620 | KC789001 | KC755757 | KC788875 | M | UWBM |
| UWBM 78780 | FULMR012-10 | *F. glacialis* | KC755455 | KC755595 | KC788985 | KC755740 | KC788866 | M | UWBM |
| UWBM 78781 | FULMR013-10 | *F. glacialis* | KC755456 | KC755596 | KC788986 | KC755741 | KC788867 | M | UWBM |
| UWBM 78782 | FULMR014-10 | *F. glacialis* | KC755457 | KC755597 | KC788987 | KC755742 | KC788868 | M | UWBM |
| UWBM 78783 | FULMR015-10 | *F. glacialis* | KC755458 | KC755598 | KC788988 | KC755743 | KC788869 | M | UWBM |
| USNM 638824 | FULMR001-10 | *F. glacialis* | KC755430 | KC755570 | KC788960 | KC755715 | KC788842 | M | USNM |
| USNM 638825 | FULMR002-10 | *F. glacialis* | KC755431 | KC755571 | KC788961 | KC755716 | KC788843 | M | USNM |
| USNM 638826 | FULMR003-10 | *F. glacialis* | KC755432 | KC755572 | KC788962 | KC755717 | KC788844 | M | USNM |
| USNM 638827 | FULMR004-10 | *F. glacialis* | KC755433 | KC755573 | KC788963 | KC755718 | KC788845 | M | USNM |
| USNM 638828 | FULMR005-10 | *F. glacialis* | KC755434 | KC755574 | KC788964 | KC755719 | KC788846 | M | USNM |
| USNM 623298 | FULMR006-10 | *F. glacialis* | KC755435 | KC755575 | KC788965 | KC755720 | KC788847 | M | USNM |
| USNM 623299 | FULMR007-10 | *F. glacialis* | KC755436 | KC755576 | KC788966 | KC755721 | KC788848 | M | USNM |
| NHMO 17156 | FULMR052-10 | *F. glacialis* | KC755437 | KC755577 | KC788967 | KC755722 | KC788849 | M | NHMO |
| NHMO 17780 | FULMR053-10 | *F. glacialis* | KC755438 | KC755578 | KC788968 | KC755723 | KC788850 | M | NHMO |
| NHMO 22337 | FULMR054-10 | *F. glacialis* | KC755439 | KC755579 | KC788969 | KC755724 | KC788851 | M | NHMO |
| NHMO 22338 | FULMR055-10 | *F. glacialis* | KC755440 | KC755580 | KC788970 | KC755725 | KC788852 | M | NHMO |
| NHMO 22339 | FULMR056-10 | *F. glacialis* | KC755441 | KC755581 | KC788971 | KC755726 | KC788853 | M | NHMO |
| NHMO 22340 | FULMR057-10 | *F. glacialis* | KC755442 | KC755582 | KC788972 | KC755727 | KC788854 | M | NHMO |
| NHMO 22347 | FULMR058-10 | *F. glacialis* | KC755443 | KC755583 | KC788973 | KC755728 | KC788855 | M | NHMO |
| NHMO 22348 | FULMR059-10 | *F. glacialis* | KC755444 | KC755584 | KC788974 | KC755729 | KC788856 | M | NHMO |
| NHMO 22349 | FULMR060-10 | *F. glacialis* | KC755445 | KC755585 | KC788975 | KC755730 | KC788857 | M | NHMO |
| NHMO 22350 | FULMR061-10 | *F. glacialis* | KC755446 | KC755586 | KC788976 | KC755731 | KC788858 | M | NHMO |
| NHMO 22355 | FULMR062-10 | *F. glacialis* | KC755447 | KC755587 | KC788977 | KC755732 | n/a | M | NHMO |
| NHMO 22356 | FULMR063-10 | *F. glacialis* | KC755448 | KC755588 | KC788978 | KC755733 | KC788859 | M | NHMO |
| NHMO 22357 | FULMR064-10 | *F. glacialis* | KC755449 | KC755589 | KC788979 | KC755734 | KC788860 | M | NHMO |
| NHMO 22358 | FULMR065-10 | *F. glacialis* | KC755450 | KC755590 | KC788980 | KC755735 | KC788861 | M | NHMO |
| K03-09244-05-04 | FULMR066-10 | *F. glacialis* | KC755481 | n/a | KC789002 | KC755758 | n/a | L | CWS |
| K03-09245-05-04 | FULMR067-10 | *F. glacialis* | KC755482 | KC755621 | KC789003 | KC755759 | n/a | L | CWS |
| K03-09247-05-04 | FULMR068-10 | *F. glacialis* | KC755483 | KC755622 | KC789004 | KC755760 | n/a | L | CWS |
| K03-09248-05-04 | FULMR069-10 | *F. glacialis* | KC755484 | n/a | KC789005 | KC755761 | KC788876 | L | CWS |
| K03-09249-05-04 | FULMR070-10 | *F. glacialis* | KC755485 | KC755623 | KC789006 | KC755762 | n/a | L | CWS |
| K03-09250-05-04 | FULMR071-10 | *F. glacialis* | KC755486 | KC755624 | KC789007 | KC755763 | KC788877 | L | CWS |
| K03-09252-05-04 | FULMR072-10 | *F. glacialis* | KC755487 | n/a | KC789008 | KC755764 | KC788878 | L | CWS |
| K03-09253-05-04 | FULMR073-10 | *F. glacialis* | KC755488 | n/a | KC789009 | KC755765 | n/a | L | CWS |
| K03-09256-05-04 | FULMR074-10 | *F. glacialis* | KC755489 | KC755625 | KC789010 | KC755766 | KC788879 | L | CWS |
| K03-09257-05-04 | FULMR075-10 | *F. glacialis* | KC755490 | KC755626 | KC789011 | KC755767 | KC788880 | L | CWS |
| K03-09258-05-04 | FULMR076-10 | *F. glacialis* | KC755491 | KC755627 | KC789012 | KC755768 | KC788881 | L | CWS |
| K08-21047-00-02 | FULMR077-10 | *F. glacialis* | KC755492 | KC755628 | KC789013 | KC755769 | KC788882 | M | CWS |
| K08-21048-00-02 | FULMR078-10 | *F. glacialis* | KC755493 | KC755629 | KC789014 | KC755770 | KC788883 | M | CWS |
| K08-21049-00-02 | FULMR079-10 | *F. glacialis* | KC755494 | KC755630 | KC789015 | KC755771 | KC788884 | M | CWS |
| K08-21050-00-02 | FULMR080-10 | *F. glacialis* | KC755495 | KC755631 | KC789016 | KC755772 | KC788885 | M | CWS |
| K08-21051-00-02 | FULMR081-10 | *F. glacialis* | KC755496 | KC755632 | KC789017 | KC755773 | KC788886 | M | CWS |
| K08-21052-00-02 | FULMR082-10 | *F. glacialis* | KC755497 | KC755633 | KC789018 | KC755774 | KC788887 | M | CWS |
| K08-21053-00-02 | FULMR083-10 | *F. glacialis* | KC755498 | KC755634 | KC789019 | KC755775 | KC788888 | M | CWS |
| K08-21054-00-02 | FULMR084-10 | *F. glacialis* | KC755499 | KC755635 | KC789020 | KC755776 | KC788889 | M | CWS |
| K08-21055-00-02 | FULMR085-10 | *F. glacialis* | KC755500 | KC755636 | KC789021 | KC755777 | KC788890 | M | CWS |
| K08-21056-00-02 | FULMR086-10 | *F. glacialis* | KC755501 | KC755637 | KC789022 | KC755778 | KC788891 | M | CWS |
| K08-21057-00-02 | FULMR087-10 | *F. glacialis* | KC755502 | KC755638 | KC789023 | KC755779 | KC788892 | M | CWS |
| K08-21058-00-02 | FULMR088-10 | *F. glacialis* | KC755503 | KC755639 | KC789024 | KC755780 | KC788893 | M | CWS |
| L93-60829-03-01 | FULMR089-10 | *F. glacialis* | KC755504 | KC755640 | KC789025 | KC755781 | KC788894 | M | CWS |
| L93-60830-03-01 | FULMR090-10 | *F. glacialis* | KC755505 | KC755641 | KC789026 | KC755782 | KC788895 | M | CWS |
| L93-60831-03-01 | FULMR091-10 | *F. glacialis* | KC755506 | KC755642 | KC789027 | KC755783 | KC788896 | M | CWS |
| L93-60832-02-01 | FULMR092-10 | *F. glacialis* | KC755507 | KC755643 | KC789028 | KC755784 | KC788897 | M | CWS |
| L93-60833-03-01 | FULMR093-10 | *F. glacialis* | KC755508 | KC755644 | KC789029 | KC755785 | KC788898 | M | CWS |
| L93-60834-03-01 | FULMR094-10 | *F. glacialis* | KC755509 | KC755645 | KC789030 | KC755786 | KC788899 | M | CWS |
| L93-60835-03-01 | FULMR095-10 | *F. glacialis* | KC755510 | KC755646 | KC789031 | KC755787 | KC788900 | M | CWS |
| L93-60837-02-01 | FULMR096-10 | *F. glacialis* | KC755511 | KC755647 | KC789032 | KC755788 | KC788901 | M | CWS |
| L93-60838-03-01 | FULMR097-10 | *F. glacialis* | KC755512 | KC755648 | KC789033 | KC755789 | KC788902 | M | CWS |
| K03-10217-00-03 | FULMR098-10 | *F. glacialis* | KC755513 | KC755649 | KC789034 | KC755790 | KC788903 | L | CWS |
| K03-10218-00-03 | FULMR099-10 | *F. glacialis* | KC755514 | KC755650 | KC789035 | KC755791 | KC788904 | L | CWS |
| K03-10219-00-03 | FULMR100-10 | *F. glacialis* | KC755515 | KC755651 | KC789036 | KC755792 | KC788905 | L | CWS |
| K03-10220-00-03 | FULMR101-10 | *F. glacialis* | KC755516 | KC755652 | KC789037 | KC755793 | KC788906 | L | CWS |
| K03-10221-00-03 | FULMR102-10 | *F. glacialis* | KC755517 | KC755653 | KC789038 | KC755794 | KC788907 | L | CWS |
| K03-10222-00-03 | FULMR103-10 | *F. glacialis* | KC755518 | KC755654 | KC789039 | KC755795 | KC788908 | L | CWS |
| K03-10223-00-03 | FULMR104-10 | *F. glacialis* | KC755410 | KC755550 | KC788940 | KC755695 | KC788823 | L | CWS |
| K03-10224-00-03 | FULMR105-10 | *F. glacialis* | KC755519 | KC755655 | KC789040 | KC755796 | KC788909 | L | CWS |
| K03-10225-00-03 | FULMR106-10 | *F. glacialis* | KC755520 | KC755656 | KC789041 | KC755797 | KC788910 | L | CWS |
| K03-10226-00-03 | FULMR107-10 | *F. glacialis* | KC755521 | KC755657 | KC789042 | KC755798 | KC788911 | L | CWS |
| K03-10227-00-03 | FULMR108-10 | *F. glacialis* | KC755522 | KC755658 | KC789043 | KC755799 | KC788912 | L | CWS |
| K03-10228-00-03 | FULMR109-10 | *F. glacialis* | KC755523 | KC755659 | KC789044 | KC755800 | KC788913 | L | CWS |
| K03-10229-00-03 | FULMR110-10 | *F. glacialis* | KC755524 | KC755660 | KC789045 | KC755801 | KC788914 | L | CWS |
| K03-10230-00-03 | FULMR111-10 | *F. glacialis* | KC755525 | KC755661 | KC789046 | KC755802 | KC788915 | L | CWS |
| K03-10231-00-03 | FULMR112-10 | *F. glacialis* | KC755526 | KC755662 | KC789047 | KC755803 | KC788916 | L | CWS |
| K03-10232-00-03 | FULMR113-10 | *F. glacialis* | KC755527 | KC755663 | KC789048 | KC755804 | KC788917 | L | CWS |
| K03-10233-00-03 | FULMR114-10 | *F. glacialis* | KC755528 | KC755664 | KC789049 | KC755805 | KC788918 | L | CWS |
| UAM 19090 | FULMR115-11 | *F. glacialis* | KC755529 | KC755665 | KC789050 | KC755806 | KC788919 | M | UAM |
| UAM 19091 | FULMR116-11 | *F. glacialis* | KC755530 | KC755666 | KC789051 | KC755807 | KC788920 | M | UAM |
| UAM 19125 | FULMR117-11 | *F. glacialis* | KC755531 | KC755667 | KC789052 | KC755808 | KC788921 | M | UAM |
| UAM 19507 | FULMR118-11 | *F. glacialis* | KC755532 | KC755668 | KC789053 | KC755809 | KC788922 | M | UAM |
| UAM 19956 | FULMR119-11 | *F. glacialis* | KC755533 | KC755669 | KC789054 | KC755810 | KC788923 | M | UAM |
| F198-07 | FULMR123-11 | *F. glacialis* | KC755534 | KC755670 | KC789055 | KC755811 | KC788924 | M | ANM |
| F199-07 | FULMR124-11 | *F. glacialis* | KC755535 | KC755671 | KC789056 | KC755812 | KC788925 | M | ANM |
| ANM 2138 | FULMR125-11 | *F. glacialis* | KC755536 | KC755672 | KC789057 | KC755813 | KC788926 | M | ANM |
| F201-07 | FULMR126-11 | *F. glacialis* | KC755537 | KC755673 | KC789058 | KC755814 | KC788927 | M | ANM |
| F202-07 | FULMR127-11 | *F. glacialis* | KC755538 | KC755674 | KC789059 | KC755815 | KC788928 | M | ANM |
| ANM 2139 | FULMR128-11 | *F. glacialis* | KC755539 | KC755675 | KC789060 | KC755816 | KC788929 | M | ANM |
| ANM 2140 | FULMR129-11 | *F. glacialis* | KC755540 | KC755676 | KC789061 | KC755817 | KC788930 | M | ANM |
| ANM 2141 | FULMR130-11 | *F. glacialis* | KC755541 | KC755677 | KC789062 | KC755818 | KC788931 | M | ANM |
| ANM 2142 | FULMR132-11 | *F. glacialis* | KC755542 | KC755679 | KC789064 | KC755819 | KC788933 | M | ANM |
| F208-07 | FULMR133-11 | *F. glacialis* | KC755543 | KC755680 | KC789065 | KC755820 | KC788934 | M | ANM |
| F209-07 | FULMR134-11 | *F. glacialis* | KC755459 | KC755599 | KC788989 | KC755744 | KC788870 | M | ANM |
| F210-07 | FULMR135-11 | *F. glacialis* | KC755460 | KC755600 | KC788990 | KC755745 | KC788871 | M | ANM |
| ANM 2143 | FULMR136-11 | *F. glacialis* | KC755461 | KC755601 | KC788991 | KC755746 | KC788872 | M | ANM |
| F212-07 | FULMR137-11 | *F. glacialis* | KC755462 | KC755602 | KC788992 | KC755747 | KC788873 | M | ANM |
| ANM 2144 | FULMR138-11 | *F. glacialis* | KC755463 | KC755603 | KC788993 | KC755748 | KC788874 | M | ANM |
| F214-07 | FULMR139-11 | *F. glacialis* | KC755464 | KC755604 | KC788994 | KC755749 | n/a | M | ANM |
| MACN-Or-ct 2330 | KAARG258-07 | *F. glacialoides* | KC755544 | KC755681 | KC789066 | KC755821 | KC788935 | M | MACN |
| MACN-Or-ct 3610 | KPARG309-08 | *F. glacialoides* | KC755545 | KC755682 | KC789067 | KC755822 | KC788936 | M | MACN |
| UWBM 76033 | FULMR121-11 | *M. giganteus* | KC755546 | n/a | KC789068 | KC755823 | KC788937 | M | UWBM |
| UWBM 80999 | FULMR122-11 | *M. halli* | KC755547 | n/a | KC789069 | KC755824 | KC788938 | M | UWBM |
| UWBM 61674 | FULMR120-11 | *P. nivea* | KC755548 | n/a | KC789070 | KC755825 | n/a | M | UWBM |
| UWBM 81012 | FULMR016-10 | *T. antarctica* | KC755549 | n/a | KC789071 | KC755826 | KC788939 | M | UWBM |

*M, muscle sample; L, liver section

†AMNH, American Museum of Natural History; ANM, Agder Naturmuseum; CWS, Canadian Wildlife Service, Specimen Bank; MACN, Museo Argentino de Ciencias Naturales "Bernardino Rivadavia"; NHMO, Natural History Museum, University of Oslo; UAM, University of Alaska Museum, Fairbanks; USNM, Smithsonian Institution, National Museum of Natural History; UWBM, Burke Museum of Natural History and Culture
